# Disseminated Gonococcal Infection Presenting as Septic Wrist Arthritis and Endocarditis

**DOI:** 10.7759/cureus.104940

**Published:** 2026-03-09

**Authors:** Layan Abu Alya, Genan Arman, Vidya Sundareshan

**Affiliations:** 1 General Practice, An-Najah National University, Nablus, PSE; 2 Internal Medicine, Southern Illinois University School of Medicine, Springfield, USA; 3 Infectious Diseases, Southern Illinois University School of Medicine, Springfield, USA

**Keywords:** case report, disseminated gonococcal infection, infective endocarditis, neisseria gonorrhoeae, septic arthritis

## Abstract

Disseminated gonococcal infection (DGI) is an uncommon manifestation of *Neisseria gonorrhoeae* infection and may rarely involve the endocardium. We report the case of a 48-year-old man who presented with progressive right wrist pain and swelling, back pain, intermittent fever, weight loss, and diarrhea. MRI demonstrated inflammatory changes involving the L3-L4 facet joints and sacroiliac joints. Culture from wrist aspiration grew *N. gonorrhoeae*, whereas blood cultures remained negative. Additional infectious evaluation showed a negative HIV screen, nonreactive syphilis testing, a negative hepatitis panel, and a negative urine nucleic acid amplification test/PCR for both *Chlamydia trachomatis* and *N. gonorrhoeae*. Transthoracic and transesophageal echocardiography demonstrated tricuspid vegetation and indeterminate bicuspid aortic valve involvement, while CT angiography confirmed tricuspid vegetations and showed an irregular ascending aortic plaque/atheroma without definite aortic valve vegetation. The patient underwent operative irrigation and debridement of the wrist and completed a six-week IV antibiotic course after multidisciplinary evaluation. This case highlights that DGI may present without classic mucocutaneous findings or positive mucosal testing and that negative blood cultures do not exclude disseminated disease or endocardial involvement.

## Introduction

Gonorrhea is a common sexually transmitted infection caused by *Neisseria gonorrhoeae*. The World Health Organization estimated 82.4 million new gonorrhea infections globally in 2020 among adults aged 15-49 years [1]. Although most infections involve genital, rectal, or pharyngeal mucosa, dissemination can occur from a mucosal infection to the bloodstream and distant organs [2,3]. Disseminated gonococcal infection (DGI) most commonly presents with dermatitis, tenosynovitis, migratory polyarthralgia, or septic arthritis; however, mucosal symptoms may be absent, and urogenital or anorectal infection may be asymptomatic. Rare but severe complications include endocarditis and meningitis [2]. Although DGI prevalence can vary from 0.5% to 3%, it can be confused with rheumatologic diseases [4]. Here, we describe an atypical presentation of DGI with culture-confirmed septic wrist arthritis, negative blood cultures, negative urine nucleic acid amplification test (NAAT)/PCR, and echocardiographic evidence of tricuspid endocardial involvement with indeterminate aortic findings.

## Case presentation

A 48-year-old man with a history of heavy alcohol use and smoking presented to the emergency department with right wrist swelling and pain for two days. His symptoms had started approximately two weeks earlier with left-sided neck pain, facial swelling that later subsided, and lower back pain, for which he had initially been discharged with muscle relaxants and analgesics. His past medical history was notable for pulmonary nodules detected in 2010 that were attributed to pigeon exposure. At that time, he had been hospitalized for two weeks but was lost to follow-up. He also had poor dentition, requiring multiple extractions, and had not seen a dentist in several years. He worked as a mechanic and had no significant family medical history.

Two days later, his back pain worsened to the point of immobility, prompting an MRI, which revealed severe L3-L4 facet arthritis with joint effusion, paraspinal muscle edema, and sacroiliac joint fluid. He was given a course of steroids, which provided partial relief, but the symptoms persisted. He also reported worsening right wrist pain, with swelling that had slightly improved. In addition, he described a subjective fever two days earlier, night sweats for three weeks, hot and cold flashes, and an unintentional 25-lb weight loss over the preceding three months. Although he reported a good appetite, he had difficulty eating because of wrist pain. He also endorsed intermittent diarrhea for the past three weeks, with his last bowel movement occurring four days earlier. He denied chest pain, shortness of breath, headache, visual changes, rash, abdominal pain, nausea, vomiting, trauma, and IV drug use.

On examination, he was afebrile and hemodynamically stable. The right wrist demonstrated tenderness and decreased range of motion. Bedside aspiration of the wrist was performed because of concern for septic arthritis; the Gram stain was negative, and the sample volume was insufficient for cell count. He was admitted for further evaluation. Initial laboratory studies and cultures were unrevealing. Chest radiography showed pulmonary nodules, raising concern for a granulomatous or fungal process. Autoimmune and fungal studies, including urine Histoplasma testing, were negative. Chest CT demonstrated findings consistent with prior granulomatous disease. Rheumatology and pulmonology were consulted, and empiric antibiotics were initially withheld pending further evaluation.

The patient later developed a fever, and culture from the wrist aspiration grew *N. gonorrhoeae*. He was started on ceftriaxone, doxycycline, and vancomycin. Orthopedic surgery performed operative irrigation and debridement of the right wrist. Infectious diseases was consulted, and the infectious workup was expanded. Additional infectious screening demonstrated a negative HIV screen; a nonreactive syphilis test; a negative hepatitis panel, including hepatitis B surface antigen, hepatitis B surface antibody, and hepatitis C antibody; and a negative urine NAAT/PCR for both *Chlamydia trachomatis *and *N. gonorrhoeae*. Blood cultures remained negative throughout hospitalization.

**Figure 1:**
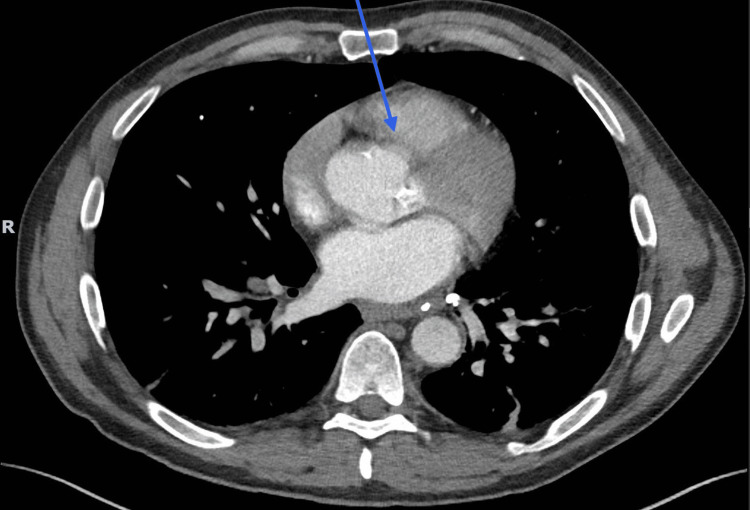
CTA A CTA demonstrated tricuspid vegetations and an irregular atherosclerotic plaque/atheroma in the ascending aorta. Superimposed vegetation involving the aortic plaque could not be excluded. No definite vegetation was visualized on the bicuspid aortic valve on the CTA. CTA, CT angiogram

Transthoracic echocardiography showed a 1.3 × 1.8 cm tricuspid valve lesion suspicious for vegetation, along with additional thickening of the aortic and mitral valves. Transesophageal echocardiography demonstrated a 1.44 × 0.76 cm mobile vegetation on the anterior leaflet of the tricuspid valve, a bicuspid aortic valve with moderate calcification in which vegetation could not be excluded, and a small mobile plaque/atheroma involving the ascending aorta. CT angiography confirmed tricuspid vegetations and demonstrated an irregular atherosclerotic plaque in the ascending aorta; however, vegetation could not be ruled out. No vegetation was seen on the bicuspid aortic valve on CT angiography, as shown in Figure 1. Cardiothoracic surgery was consulted, and Panorex imaging revealed extensive dental caries. The recommendation was a six-week course of IV antibiotics, followed by repeat echocardiography and annual aortic surveillance.

The patient remained afebrile and hemodynamically stable for the remainder of his hospitalization. No additional sites of infection were identified. He was discharged in stable condition with planned follow-up with infectious disease, cardiology, and cardiothoracic surgery.

## Discussion

This case illustrates an atypical presentation of DGI. Although the classic arthritis-dermatitis syndrome includes tenosynovitis, migratory polyarthralgia, and skin lesions, DGI can also present as isolated or oligoarticular septic arthritis, and mucosal symptoms may be absent [2,3,5]. In our patient, the absence of mucocutaneous findings, the negative urine NAAT/PCR, and the lack of a clearly documented contributory sexual history lowered the initial suspicion for gonococcal infection. However, culture from the wrist aspiration confirmed *N. gonorrhoeae*. Atypical presentations that mimic rheumatologic disease have also been described in the literature [4].

The diagnostic course was further complicated by persistently negative blood cultures. This does not exclude DGI. Current CDC guidance recommends obtaining NAAT and culture specimens from exposed urogenital and extragenital sites, as well as from disseminated sites such as synovial fluid, blood, or skin lesions when DGI is suspected [2]. In our patient, the microbiologic diagnosis rested on culture of the wrist aspirate, while urine NAAT/PCR, HIV screening, syphilis testing, hepatitis studies, and blood cultures were negative. This pattern highlights the importance of maintaining suspicion for DGI even when initial mucosal- or blood-based testing is unrevealing [2,5].

Cardiac imaging in this case required careful interpretation. Transthoracic and transesophageal echocardiography demonstrated tricuspid vegetation, whereas the aortic findings remained indeterminate. Transesophageal echocardiography raised concern for possible aortic valve involvement in the setting of a calcified bicuspid aortic valve, but CT angiography did not demonstrate definite aortic valve vegetation and instead showed an irregular ascending aortic plaque/atheroma. Accordingly, this case is best interpreted as tricuspid endocardial involvement with indeterminate aortic findings rather than definite aortic valve endocarditis. Gonococcal endocarditis remains a rare but severe manifestation of DGI and has been associated with substantial valvular destruction in previous reports [6-9].

Management of cardiovascular DGI should be individualized and multidisciplinary. CDC recommends ceftriaxone-based parenteral therapy for gonococcal meningitis or endocarditis, with additional anti-chlamydia therapy only if *C. trachomatis* infection has not been excluded [2]. In this case, doxycycline was included initially as empiric coverage before coinfection had been excluded, whereas vancomycin represented empiric early coverage rather than directed therapy against *N. gonorrhoeae*. CDC also notes that treatment duration for DGI involving the cardiovascular system has not been systematically studied and that parenteral therapy for endocarditis should continue for more than four weeks in consultation with infectious disease specialists [2]. In our patient, a six-week IV regimen was selected after multidisciplinary consultation with infectious disease, cardiology, and cardiothoracic surgery.

A limitation of this report is that a complete sexual exposure history, gonococcal antimicrobial susceptibility data, and long-term post-discharge cardiac imaging outcomes were not fully available in the accessible record. These missing data limit definitive interpretation of the aortic abnormality and microbiologic characterization of the isolate.

## Conclusions

DGI can present with nonspecific musculoskeletal symptoms and, less commonly, progress to endocardial involvement. In this case, culture-confirmed monoarticular septic arthritis of the wrist occurred alongside blood culture-negative tricuspid endocardial involvement with indeterminate aortic findings, without mucocutaneous stigmata and without a clearly documented contributory sexual history. Early arthrocentesis with operative irrigation and debridement, timely echocardiographic assessment, and multidisciplinary management involving infectious disease, orthopedics, cardiology, and cardiothoracic surgery supported clinical stabilization and a six-week IV treatment course. This case underscores the need to maintain a high index of suspicion for DGI in unexplained septic arthritis and to consider early echocardiographic evaluation when systemic features are present.
